# ICTV Virus Taxonomy Profile: Sedoreoviridae 2022

**DOI:** 10.1099/jgv.0.001782

**Published:** 2022-10-10

**Authors:** Jelle Matthijnssens, Houssam Attoui, Krisztián Bányai, Corina P. D. Brussaard, Pranav Danthi, Mariana del Vas, Terence S. Dermody, Roy Duncan, Qín Fāng (方勤), Reimar Johne, Peter P. C. Mertens, Fauziah Mohd Jaafar, John T. Patton, Takahide Sasaya (笹谷孝英), Nobuhiro Suzuki (鈴木信弘), Taiyun Wei (魏太云)

**Affiliations:** 1 University of Leuven, Leuven, Belgium; 2 National Institute for Agricultural Research (INRA), Maisons Alfort, France; 3 Veterinary Medical Research Institute, Budapest, Hungary; 4 NIOZ Royal Netherlands Institute for Sea Research & University of Utrecht, Texel, The Netherlands; 5 Indiana University, Bloomington, USA; 6 Instituto de Agrobiotecnología y Biología Molecular (IABIMO), Buenos Aires, Argentina; 7 University of Pittsburgh School of Medicine, Pittsburgh, Pennsylvania, USA; 8 Dalhousie University, Halifax, Nova Scotia, Canada; 9 Wuhan Institute of Virology, Wuhan, PR China; 10 German Federal Institute for Risk Assessment, Berlin, Germany; 11 University of Nottingham, Nottingham, UK; 12 Ecole Nationale Vétérinaire d’Alfort, Maisons Alfort, France; 13 National Agriculture and Food Research Organization, Fukuyama, Japan; 14 Okayama University, Kurashiki, Japan; 15 Fujian Agriculture and Forestry University, Fuzhou, PR China

**Keywords:** ICTV Report, taxonomy, *Sedoreoviridae*, *Reovirales*

## Abstract

*Sedoreoviridae* is a large family of icosahedral viruses that are usually regarded as non-enveloped with segmented (10–12 linear segments) dsRNA genomes of 18–26 kbp. Sedoreovirids have a broad host range, infecting mammals, birds, crustaceans, arthropods, algae and plants. Some of them have important pathogenic potential for humans (e.g. rotavirus A), livestock (e.g. bluetongue virus) and plants (e.g. rice dwarf virus). This is a summary of the ICTV Report on the family *Sedoreoviridae*, which is available at ictv.global/report/sedoreoviridae.

## Virion

**Fig. 1.**
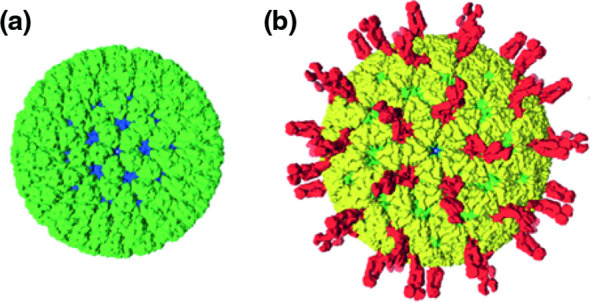
Sedoreovirid particle structure (e.g. rotavirus A). Particles are coloured based on functional similarity (blue – core shell, green – middle layer, yellow – outer capsid, red – membrane penetration and receptor binding). (a) Subviral particle. (b) Virion. The diameter of the mature virion is roughly 80 nm (excluding spikes). Adapted from [7].

Sedoreovirid particles are icosahedral (Table 1). The protein capsid is organized as 1–3 concentric layers of capsid proteins, with an overall diameter of 60–100 nm [1]. Members of the family *Sedoreoviridae* lack large surface projections on the subviral particle, giving them an almost spherical or ‘smooth’ appearance (Fig. 1), in contrast to members of the family *Spinareoviridae,* which have spikes or turrets at the 12 icosahedral vertices.

**Table 1.** Characteristics of members of the family *Sedoreoviridae*

Example:	rotavirus A RVA/Simian-tc/ZAF/SA11-H96/1958/G3P5B[2] (Seg1: DQ838640; Seg2: DQ838635; Seg3: DQ838645; Seg4: DQ841262; Seg5: DQ838599; Seg6: DQ838650; Seg7: DQ838610; Seg8: DQ838615; Seg9: DQ838620; Seg10: DQ838625; Seg11: DQ838630), species *Rotavirus A*, genus *Rotavirus*
Virion	Non-enveloped, icosahedral, 60–100 nm virions composed of 1–3 concentric capsid proteins layers
Genome	18–26 kbp of segmented linear dsRNA, with each of the 10–12 segments ranging from 0.6 to 5.8 kbp
Replication	Replication occurs in the cytoplasm in electron-dense structures called viroplasms or virus inclusion bodies
Translation	From full-length transcribed mRNAs, which possess a 5′-terminal cap but no poly(A)-tail
Host range	Mammals, birds, crustaceans, arthropods, algae and plants
Taxonomy	Realm *Riboviria,* kingdom *Orthornavirae*, phylum *Duplornaviricota,* class *Resentoviricetes,* order *Reovirales*: >5 genera and >35 species

## Genome

Sedoreovirids contain 10–12 segments of linear dsRNA comprising 18–26 kbp in total, with individual segments ranging from 0.6 to 5.8 kbp. The positive-sense strands of each duplex are modified with a 5′-terminal type 1 cap structure but no 3′-poly(A) tail. The viral RNAs are mostly monogenic with relatively short 5′- and 3′-non-coding regions, although some segments have a second or third functional ORF [2].

## Replication

Virus cell entry varies between genera but usually results in loss of outer-capsid components. The resulting transcriptionally active particles are released into the cytoplasm. The 5′-capped mRNAs are synthesized by structural enzymatic components of the particle and are released through pores at the icosahedral apices of the virion into the cytoplasm. Viroplasms, also known as virus inclusion bodies, are distributed throughout the cytoplasm. These neo-organelles are sites of viral mRNA synthesis, genome replication and particle assembly [3]. Sets of a single copy of each capped mRNA are incorporated into progeny virus particles [4]. These mRNAs serve as templates for negative-sense strand synthesis, thereby reconstituting genomic encapsidated dsRNAs. Progeny virions are released without compromising cell viability (e.g. budding) or following cell lysis, depending on the cell type [1,5].

## Taxonomy

**Fig. 2.**
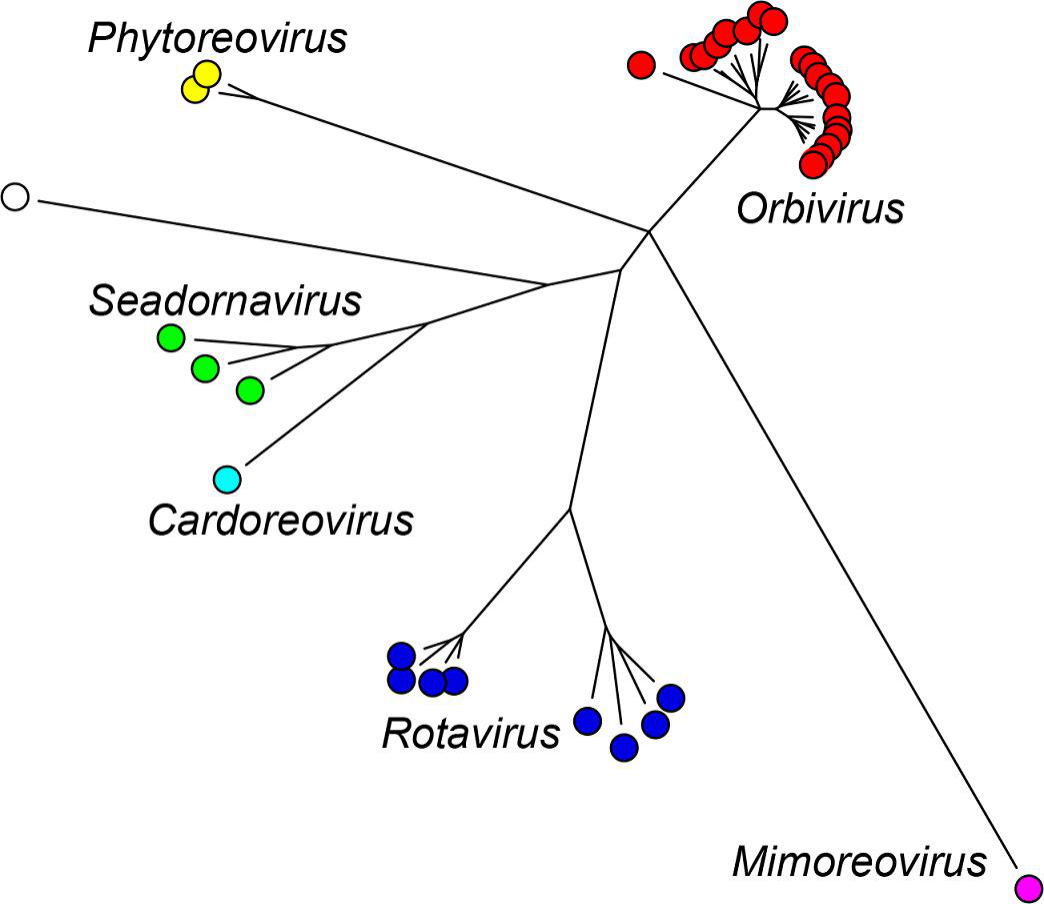
Sedoreovirus phylogeny based on RNA-directed RNA polymerase amino acid sequences. For details see full ICTV Report.

Current taxonomy: ictv.global/taxonomy. The family *Sedoreoviridae* includes several genera (Fig. 2) and >35 species [6] of viruses infecting mammals, birds, crustaceans, arthropods, algae and plants. The number of genome segments (10–12) is characteristic of viruses within a single genus. Other factors distinguishing different genera are host (and vector) range, disease signs and capsid structure. The amino acid sequence of the relatively conserved RNA-directed RNA polymerase can be used for comparison across taxonomic boundaries. Among the members of a species, protein and RNA sequences are relatively conserved, being serologically cross-reactive and including specific RNA packaging signals. This high degree of functional and structural compatibility allows viable progeny virus strains to be generated by reassortment between viruses of the same species.

## Resources

Full ICTV Report on the family *Sedoreoviridae*: ictv.global/report/sedoreoviridae
